# To Possess or to Experience: Personal Values and Sociocultural Context Moderated Age Differences in Purchase Evaluations

**DOI:** 10.1002/ijop.70147

**Published:** 2025-12-11

**Authors:** Haowei Peng, Tianyuan Li

**Affiliations:** ^1^ School of Humanities and Social Science The Chinese University of Hong Kong Shenzhen China; ^2^ Harvard Graduate School of Education Harvard University Cambridge Massachusetts USA

**Keywords:** conservation, experiential purchase, material purchase, openness, self‐transcendence

## Abstract

Purchase decisions have important implications for consumers' well‐being. Age‐related motivation changes may affect purchase preferences, while personal values and sociocultural context can moderate the age differences. This study investigates age‐related differences in experiential versus material purchase evaluations and the moderating effects of personal values and sociocultural contexts with participants aged 20–78 years from the United States (*N* = 201) and China (*N* = 212). A significant country × personal value × age three‐way interaction on purchase evaluation was found. Age was negatively related to experiential purchase preference for Chinese participants with lower self‐transcendence or openness‐to‐change values. Such a relationship became weaker or even positive for Chinese participants with higher self‐transcendence or openness‐to‐change values. However, no age‐related main effects or interactions were significant in American participants. The study provides insights into age‐related differences in consumers' purchase preferences and highlights the critical roles of personal values and sociocultural contexts.

## 1 Introduction

The investigations of experiential purchases' advantages on happiness brought attention to the distinction between experiential and material purchases (van Boven and Gilovich 2003). Specifically, experiential purchases refer to the consumption of events and experiences, while material purchases are about tangible goods that one could possess (van Boven and Gilovich 2003). The finding that experiential purchases yield greater happiness compared to material purchases has been replicated in multiple studies (e.g., Carter and Gilovich 2010; Pchelin and Howell 2014; Thomas and Millar 2013).

To understand the different effects brought by experiential and material purchases, researchers examined consumers' evaluations of these two types of purchases and identified three main aspects that might explain the more beneficial effects brought by experiential purchases (for a review, see Gilovich and Gallo 2020). First, compared to material purchases, consumers reported experiential purchases to be more helpful in promoting social connections. The purchased experiences can convey certain messages to one's social network, arouse a sense of social connectedness and help build and maintain high‐quality interpersonal relationships (Caprariello and Reis 2013; Kumar and Gilovich 2015; Yamaguchi et al. 2016). Second, experiential purchases are perceived to be more useful for supporting one's identity. While materialism is negatively related to consumers' self‐concept clarity (Noguti and Bokeyar 2014), people perceive experiential purchases to resonate with their true selves and reflect their identity, as well as help them move towards their ideal selves by enriching their abilities and contributing to self‐actualisation (Carter and Gilovich 2012; Kim et al. 2016; Moldes et al. 2019; van Boven and Gilovich 2003). Finally, the enjoyment derived from experiential purchases is less likely to be affected by social comparisons (Carter and Gilovich 2010; Howell and Hill 2009). Consumers tend to find more satisfaction from experiential purchases, as experiences are evaluated more on their own terms rather than being subject to comparison with other people's purchases based on monetary value (Gilovich and Gallo 2020).

Although the experiential advantages were widely discussed, questions remain regarding the universality of purchase evaluation preference across different populations and contexts. Various individual factors have been explored to understand how experiential and material purchases were evaluated, such as gender (Carter and Gilovich 2012), personality (Zhang et al. 2014) and socioeconomic status (SES; Thomas and Millar 2013; van Boven and Gilovich 2003). Age is also an essential factor that may influence purchase evaluations but is understudied in the current literature. With increasing age, people tend to prioritise emotionally meaningful goals (Fung and Carstensen 2004). The shifted motivation could substantially change their purchase evaluations. Facing the unprecedented population ageing trend, older adults' purchasing power is growing (Hettich et al. 2018). It is of high importance to understand the age‐related differences in purchase preferences, which provide valuable insights into older consumers' purchase decisions.

### 1.1 Age Differences in Purchase Evaluations

According to the socioemotional selectivity theory (Carstensen 2021), people become more conscious of the time constraints as they age. While young adults emphasise future‐oriented goals, older adults prioritise emotionally meaningful goals that emphasise close relationships with family and friends (Fung and Carstensen 2004). The shift towards emotionally meaningful goals and away from future‐oriented goals may affect older adults' purchase evaluations. There are two competing possibilities regarding the age differences in preference for experiential purchases.

On the one hand, older age can be related to a stronger preference for experiential purchases. Experiential purchases can bring social benefits by fostering mutual affection and social relationships through shared activities (Caprariello and Reis 2013), which may help older adults fulfil their need to strengthen social connections and attain emotional fulfilment. Moreover, according to the terror management theory (Greenberg et al. 1986), when threatened by death, individuals tend to seek psychological comfort that provides them with a sense of continuity beyond their physical existence. Experiential purchases contribute to more meaningful memories, one's identity and stronger social connectedness, enhancing the overall sense of meaning in life (Gupta et al. 2024). Thus, older adults, who can perceive death's increasing proximity (Maxfield and Bevan 2019), may attach greater significance to experiential purchases and rate them more favourably.

On the other hand, older age may be related to less preference for experiential purchases. Experiential products can be useful for self‐improvement and establishing new social connections, which align closely with young adults' personal growth needs and emphasis on future‐oriented goals (e.g., van Boven and Gilovich 2003). As older adults' priority shifts away from new social experiences and future‐oriented goals (Fung and Carstensen 2004), they may not perceive the benefits of experiential products as appealing as those of younger individuals. Thus, it is possible that older consumers would evaluate experiential purchases less favourably compared to their younger counterparts.

The competing possibilities about the age‐related differences in purchase preferences highlight the importance of empirically examining the question. Moreover, as existing evidence suggests that different factors are influencing the age‐related differences in purchase preferences in opposite directions, it indicates that there may not be a unifying pattern about how purchase preferences change with age. Instead, individual dispositions and contextual factors may moderate the relationship. Thus, in the current study, we empirically examine the age‐related differences in evaluations towards experiential versus material purchases. We also investigate how sociocultural context and personal values moderate the relationship between age and purchase preferences.

### 1.2 Personal Values and the Age Differences in Purchase Evaluations

Personal values reflect individuals' underlying motivation and prioritised needs in life, shaping their attitudes, behaviours and preferences (Schwartz 2003). Schwartz (2003, 2012) synthesises the diverse personal values into two orthogonal dimensions: self‐transcendence versus self‐enhancement (referred to as *transcendence‐enhancement* in the rest of the article) and openness‐to‐change versus conservation (referred to as *openness‐conservation* in the rest of the article). While no existing research has directly examined either the main effects of personal values on experiential versus material purchase preference or how personal values and age interact to influence such preferences, a growing body of evidence suggests that personal values are related to a variety of preferences, decisions and behavioural tendencies in consumption (e.g., Blomstervik and Olsen in press; Krystallis et al. 2012). We expect both value dimensions to be associated with experiential versus material purchase preference and to moderate age‐related differences in the preference.

First, the transcendence‐enhancement dimension manifests the contrast between power and achievement values versus universalism and benevolence values. The former emphasises the pursuit of personal growth and self‐interests, while the latter concerns the welfare and interests of others and humankind in general (Schwartz 2012). Previous research has found that self‐enhancement values are positively associated with materialism, a key driver for engaging in luxury consumption (Hudders and Pandelaere 2012; Kilbourne and LaForge 2010). Meanwhile, individuals high in self‐transcendence values care more about the impacts of their purchases on other people and the community. They are more likely to purchase organic food (Krystallis et al. 2012), support ethical and fair‐trade consumption (Doran 2009; Shaw et al. 2005) and reject genetically modified products (Honkanen and Verplanken 2004). Individuals who prioritise self‐transcendence values may view experiential purchases as a better way to cultivate positive social relationships, which aligns with their focus on social connectedness and primary concerns of others' interests (Schwartz 2012). Thus, our first hypothesis is:

Hypothesis 1 *Stronger endorsement of the self‐transcendence values is related to more favourable evaluations for experiential over material purchases*.

On the other side, the openness‐conservation dimension represents the contrast between self‐direction and stimulation values as opposed to security, conformity and tradition values. The former prioritises independent actions and readiness for exploration and novel experiences, whereas the latter underscores self‐restriction, order and resistance to change (Schwartz 2003, 2012). As the experiential purchases are more linked with novelty and exploration, which are more consistent with the needs of individuals who emphasise openness‐to‐change over conservation values (Kim et al. 2016), they may evaluate experiential purchases more favourably. Previous research has also identified that consumers who prioritise openness‐to‐change are more likely to seek novel experiences, while those who value conservation prefer familiarity (Blomstervik and Olsen in press). The second hypothesis is:

Hypothesis 2 *Stronger endorsement of the openness‐to‐change values is related to more favourable evaluations for experiential over material purchases*.

Moreover, personal values can also influence the age‐related differences in purchase preferences. As mentioned above, older age can either be related to a stronger preference for experiential purchase as the result of older adults' increasing emphasis on emotional meaningfulness or be related to less preference for experiential purchase because of older adults' reduced interest in new experiences and future‐oriented goals. Personal value can be a critical factor shaping how people's purchase preferences change with age. In particular, because of the emphasis on social connections over personal gains, individuals who emphasise self‐transcendence values may especially value the social meaningfulness of their purchases while they age, resulting in a more positive relationship between age and the preference for experiential purchases. In contrast, individuals who lean towards the self‐enhancement values may care more about personal possessions and prefer material purchases more as they age. Thus, the third hypothesis is:

Hypothesis 3 *Stronger endorsement of the self‐transcendence values is related to a more positive relationship between age and evaluation preference for experiential over material purchases*.

Similarly, because of the preference for new experiences, individuals who prioritise openness‐to‐change values may still enjoy experiential purchases while they age, resulting in a more positive relationship between age and the evaluation of experiential purchases. In contrast, individuals who prioritise conservation values might be more dedicated to routine behaviours while they age and devalue experiential purchases accordingly. The fourth hypothesis is:

Hypothesis 4 *Stronger endorsement of the openness‐to‐change values is related to a more positive relationship between age and evaluation preference for experiential over material purchases*.

### 1.3 Sociocultural Context and the Age Differences in Purchase Evaluations

When investigating age‐related differences in purchase evaluations and the potential moderating role of personal values, it is important to consider the sociocultural context as well. Although the basic value structure is universal, the prioritisation of certain values varies across cultures (Schwartz 2006). For instance, individualistic cultures like the United States tend to prioritise values related to self‐enhancement and openness‐to‐change, whereas people in collectivist countries with Confucian cultures, such as China, tend to emphasise self‐transcendence and conservation (Schwartz 2006; Schwartz and Bardi 2001). Consumers from different cultural backgrounds also tend to favour brands that align with their cultural beliefs and values (Torelli et al. 2012), as well as displaying different preferences and decision‐making styles regarding brand, price and quality (de Mooij and Hofstede 2011). Understanding cultural differences is crucial for interpreting how personal values can be related to consumer behaviour, highlighting the importance of situating the current study within specific cultural contexts.

Moreover, when investigating age‐related differences in purchase preferences, we must consider the sociocultural changes that have occurred over recent decades in different societies. For example, in developing societies like China, there have been significant economic transformations since the 1980s (Yao 2000). The rapid economic development has influenced individuals' values, beliefs and behaviours in consumption (Hung et al. 2007), such as a rising trend of materialism among Chinese young adults (Su et al. 2024), which may enlarge the age‐related differences in purchase preferences. In contrast, in developed societies like the United States, as the socioeconomic environment is relatively stable, the age‐related differences in purchase beliefs and behaviours can be less pronounced (Hung et al. 2007). Thus, the current study explores how age‐related disparities in purchase evaluations vary across different sociocultural contexts, specifically comparing China and the United States. The last hypothesis is:

Hypothesis 5 *The age‐related differences in purchase evaluations and the personal value × age interaction effects are more apparent in China compared to the United States*.

### 1.4 The Current Study

To conclude, the current study aims to investigate how age is associated with individuals' changing purchase preferences and how individual characteristics (i.e., personal values) and contextual factors (i.e., cultural context) jointly shape the age‐related changes in purchase preferences. It addresses the pressing need to understand older consumers' preferences facing the population ageing trend. It also bridges consumer research with life‐span developmental theories, Schwartz's value system and the cross‐cultural perspective, leading to a more dynamic and holistic understanding of consumer behaviours.

## 2 Methods

### 2.1 Participants and Procedure

An a priori power analysis was conducted with G*Power (Faul et al. 2007) to determine the required sample size. The country × personal value × age interaction was considered as a predictor in a multiple regression model, and a small effect size (partial *R* ^2^ = 0.015) was expected. A sample size of 408 was required to achieve 0.80 power at an *α* level of 0.05 for detecting such an effect. Thus, we aimed to recruit 204 participants in each country. A total of 234 participants from the United States were recruited from the online platform Mechanical Turk (MTurk). Thirty‐three participants were excluded because they failed to pass the quality check. The final sample consisted of 201 participants aged from 20 to 78 years old (mean_age_ = 44.29, SD_age_ = 16.37; 91 females, 110 males). The majority of participants were Caucasian (81.1%). Most of them held a bachelor's degree (74.6%), were employed full‐time (88.6%), and had an annual household income between $40,000 and $60,000 (37.8%) or $60,000 and $80,000 (22.9%). Meanwhile, 239 participants from China were recruited from Credamo. Twenty‐seven participants were excluded as they failed to pass the quality check. The final sample consisted of 212 participants aged from 18 to 67 years old (mean_age_ = 40.40, SD_age_ = 16.58; 115 females and 97 males). The majority of participants held a bachelor's degree (61.8%), were employed full‐time (55.7%), and had an annual household income between 100,001 and 200,000 RMB (38.7%) or 200,001 and 300,000 RMB (24.5%).

After granting informed consent, participants completed an online survey about purchase evaluations, personal values and other relevant information. They received either $0.5 or 8.5 RMB after completing the survey.

### 2.2 Measures

#### 2.2.1 Experiential Versus Material Purchase Evaluations

Five material items (i.e., a pair of jeans, a television set, a smartphone, a wristwatch and a diamond necklace) and five experiential items (i.e., a beach vacation, a ski pass, a meal at a nice restaurant, a concert ticket and a cruise package) were chosen based on widely adopted items in previous studies (Kim et al. 2016; Kumar and Gilovich 2015). Participants evaluated each purchase item comprehensively from five aspects adapted and derived from previous studies (Li et al. 2022; Moldes et al. 2019; Pchelin and Howell 2014; Thomas and Millar 2013; van Boven and Gilovich 2003). In particular, participants evaluated to what extent each item ‘is meaningful to your life’, ‘can help you establish new social relationships or strengthen the existing ones’, ‘can prepare you for future challenges’, ‘can express who you are’ and ‘can contribute to your happiness in life’ on a rating scale from 1 (*not at all*) to 7 (*very much*). The order of the 10 purchase items and the five evaluation questions for each item was randomised in the online survey. We first calculated the average evaluation rating for all experiential items and that for all material items. Then we calculated the final purchase evaluation score by subtracting the average rating for material items (Cronbach's *α* = 0.95) from the average rating for experiential items (Cronbach's *α* = 0.94), so higher scores indicate more favourable evaluations for the experiential purchases over the material ones.

#### 2.2.2 Personal Values

Personal values were assessed by the 21‐item Portrait Values Questionnaire (PVQ‐21; Schwartz 2003; *α* = 0.95). Each item describes a portrait of a person based on what is important to him/her. Participants rated each item on a rating scale from 1 (*very much like me*) to 6 (*not like me at all*). All ratings were first reversed so that a higher number would indicate a stronger endorsement of the statement. As recommended in previous research (Schwartz and Rubel 2005), we then calculated the ipsatised score for all items by subtracting the within‐individual mean of the 21 items for each participant from their item ratings. The ipsatised scores removed the between‐individual differences in the average item rating and better reflected individuals' relative emphasis on each value. The score for the *transcendence‐enhancement* dimension (Cronbach's *α* = 0.72) was then calculated by averaging the ratings on self‐transcendence items (e.g., ‘It's very important to him/her to help the people around him/her. He/she wants to care for their Well‐being.’) and the reversed ratings on self‐enhancement items (e.g., ‘It is very important to him/her to show his/her abilities. He/she wants people to admire what he/she does.’), with higher scores indicating stronger endorsement of self‐transcendence values over self‐enhancement ones. The score for the *openness‐conservation* dimension (Cronbach's *α* = 0.61) was calculated by averaging the ratings on openness‐to‐change items (e.g., ‘Thinking up new ideas and being creative is important to him/her. He/she likes to do things in her own original way.’) and the reversed ratings on conservation items (e.g., ‘It is important to him/her to live in secure surroundings. He/she avoids anything that might endanger his/her safety.’), with higher scores indicating stronger endorsement of openness‐to‐change values over conservation ones.

#### 2.2.3 Current and Childhood SES

Participants' current and childhood SES were measured as covariates, as previous research suggests that both current SES (Thomas and Millar 2013; van Boven and Gilovich 2003) and childhood experiences in financial adversity (Mittal and Sundie 2017) may play a significant role in shaping individuals' purchase preferences. The current SES was indicated by participants' annual household income (1 = *below $20,000* to 6 = *above $100,000* for American participants; 1 = *below 50,000 RMB* to 6 = *above 400,000 RMB* for Chinese participants) and education levels (1 = *did not attend school* to 7 = *advanced degree*). Individuals' income and education scores were first standardised within each country. The two *z*‐scores were then summed up to indicate participants' current SES, with higher scores indicating higher current SES. Participants' childhood SES was assessed by the 3‐item Perceived Wealth in Childhood Scale (Griskevicius et al. 2011; Cronbach's *α* = 0.93), which asked participants about their perceptions of SES during their upbringing (e.g., ‘My family usually had enough money for things when I was growing up.’). The items were rated on a 9‐point Likert scale (1 = *strongly disagree*, 9 = *strongly agree*). The average of the three item ratings was calculated, with higher scores indicating higher childhood SES.

### 2.3 Data Analysis

Data analyses were conducted using IBM SPSS Statistics (Version 25) and the R statistical software programme. We first examined the descriptive statistics of the American and the Chinese samples and tested whether there were significant differences between the two groups using independent‐sample *t* tests. We then conducted multigroup confirmatory factor analysis (CFA) tests to examine measurement invariance for the purchase evaluation and personal value measures across the Chinese and American samples. Next, we estimated two multiple regression models to examine how sociocultural context, personal value and age interacted to influence purchase preference. One model tested the transcendence‐enhancement dimension of personal values, and the other model tested the openness‐conservation dimension. Age and the two personal value dimensions were standardised before being entered into the model. As a significant country × personal value × age interaction was identified in both models, we further used multiple regressions to examine how each dimension of personal value moderated age differences in purchase preference in the two countries, respectively. We then conducted simple slope analyses using the PROCESS macro (Hayes 2013) to illustrate the age differences in purchase preference for individuals with varying levels of personal values in each country. In all regression models, participants' current and childhood SES were controlled.

## 3 Results

The descriptive statistics of the American and the Chinese samples were presented in Table 1. Results of the *t* tests revealed that Chinese participants (mean = 40.40, SD = 16.58) were younger than the American participants (mean = 44.29, SD = 16.37), *t*(411) = −2.40, *p* = 0.17. Chinese participants reported lower levels of education (mean = 5.37, SD = 1.30) than American participants (mean = 5.83, SD = 0.92), *t*(380.5) = −4.20, *p* < 0.001, as well as lower childhood SES (mean = 5.18, SD = 2.25) compared to their American counterparts (mean = 6.72, SD = 1.74), *t*(395.4) = −7.79, *p* < 0.001. Chinese participants reported stronger endorsement of the self‐transcendence values (mean = 0.32, SD = 0.59) compared to American participants (mean = 0.08, SD = 0.42), *t*(382.4) = 4.79, *p* < 0.001. Chinese participants also reported higher preference towards experiential purchases (mean = 0.59, SD = 1.01) than American participants (mean = 0.02, SD = 0.45), *t*(294.7) = 7.63, *p* < 0.001. The two groups did not differ significantly in other variables, *p*s > 0.54.

**TABLE 1:** Descriptive statistics of the American and the Chinese sample.

Variables	American (*n* = 201)		Chinese (*n* = 212)		*t*
	*M*	SD	*M*	SD	
Age	44.29	16.37	40.40	16.58	−2.40*
Education^a^	5.83	0.92	5.37	1.30	−4.20***
Annual household income^b^	3.42	1.23	3.34	1.22	−0.61
Current SES	0.00	1.55	0.00	1.47	0.00
Childhood SES	6.72	1.74	5.18	2.25	−7.79***
Transcendence‐enhancement	0.08	0.42	0.32	0.59	4.79***
Openness‐conservation	−0.02	0.34	−0.01	0.56	0.22
Purchase evaluations (experiential over material)	0.01	0.45	0.56	1.01	7.63***

^a^ Education was coded as 1 = did not attend school, 2 = primary school, 3 = high school, 4 = associates, 5 = some college but no degree, 6 = bachelor's degree, 7 = advanced degree.

^b^ Annual household income was coded as 1 = *below $20,000*, 2 = *$20,000–$40,000*, 3 = *$40,000–$60,000*, 4 = *$60,000–$80,000*, 5 = *$80,000–$100,000*, 6 = *above $100,000* for American participants, and 1 = *below 50,000 RMB*, 2 = *50,000–100,000 RMB*, 3 = *100,001–200,000 RMB*, 4 = *200,001–300,000 RMB*, 5 = *300,001–400,000 RMB*, 6 = *above 400,000 RMB* for Chinese participants.

* *p* < 0.05.

*** *p* < 0.001.

Multigroup CFAs were conducted to assess measurement invariance for the purchase evaluation measure and the PVQ scale across the two samples. For the purchase evaluation measure, all evaluation items for the material purchases were loaded on one factor, and those for the experiential purchases were loaded on a second factor. The configural equivalence model across the two samples demonstrated acceptable fit, *χ* ^2^(1778) = 2899.45, *p* < 0.001, CFI = 0.930, TLI = 0.904, RMSEA = 0.055, SRMR = 0.069, suggesting the structure of the two‐factor model held in both samples. We then tested metric equivalence across the two samples by constraining all factor loadings to be identical in both samples. The chi‐square difference test indicated a significant decline in model fit for the metric equivalence model, Δ*χ* ^2^(48) = 190.32, *p* < 0.001, suggesting that factor loadings of different items were not identical across the two samples. Similarly, for the PVQ scale, corresponding items for the transcendence‐enhancement and openness‐conservation dimensions were loaded on two factors, respectively. Again, the configural equivalence model across the two samples showed good fit, *χ* ^2^(88) = 127.70, *p* = 0.004, CFI = 0.977, TLI = 0.909, RMSEA = 0.047, SRMR = 0.049, supporting the two‐factor model in both samples. However, the chi‐square difference test again indicated a significant decline in model fit for the metric equivalence model, Δ*χ* ^2^(17) = 42.74, *p* < 0.001, failing to support metric equivalence across the two samples.

### 3.1 Sociocultural Context, Transcendence‐Enhancement Values and Age on Purchase Preference

Table 2 presented the multiple regression model testing how sociocultural context and the transcendence‐enhancement dimension of personal value interacted with age to influence purchase preference. There was a positive significant main effect of transcendence‐enhancement on evaluation preference towards experiential purchases, *b* = 0.20, *t* = 3.93, *p* < 0.001. There was also a significant positive age × transcendence‐enhancement interaction, *b* = 0.19, *t* = 4.19, *p* < 0.001, indicating that the association between age and preference for experiential purchases was more positive for individuals with stronger endorsement of the self‐transcendence values. Most importantly, supporting Hypothesis 5, there was a significant country × age × transcendence‐enhancement interaction, *b* = −0.17, *t* = −1.97, *p* = 0.049, indicating that sociocultural context further moderated the age × transcendence‐enhancement interaction.

**TABLE 2:** Country × age × transcendence‐enhancement interaction effects on purchase preference.

Variables	*B*	SE	*t*	*p*
Constant	0.47	0.06	8.33	0.000
Current SES	0.13	0.04	3.48	0.001
Childhood SES	0.02	0.04	0.49	0.624
Age	−0.23	0.05	−4.27	0.000
Country (0 = China, 1 = United States)	−0.41	0.08	−4.87	0.000
Transcendence‐enhancement	0.20	0.05	3.93	0.000
Country × age	0.23	0.08	2.94	0.004
Country × transcendence‐enhancement	0.03	0.10	0.28	0.784
Age × transcendence‐enhancement	0.19	0.05	4.19	0.000
Country × age × transcendence‐enhancement	−0.17	0.08	−1.97	0.049

*Note:* Age and transcendence‐enhancement were standardised.

**FIGURE 1:**
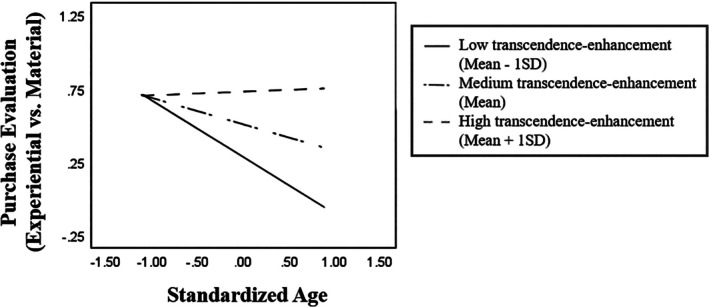
Simple slope analyses illustrating the age × transcendence‐enhancement interaction on purchase preference in Chinese participants.

Thus, we examined how transcendence‐enhancement values interacted with age to influence purchase preference in Chinese and American participants, respectively (see Table 3). For Chinese participants, age had a significant negative main effect (*b* = −0.22, *t* = −3.19, *p* = 0.002), and transcendence‐enhancement values had a significant positive main effect (*b* = 0.20, *t* = 3.10, *p* = 0.002) on purchase preference. More importantly, the age × transcendence‐enhancement interaction was significant, *b* = 0.18, *t* = 3.25, *p* = 0.001. Simple slope analyses (see Figure 1) suggest that age was significantly related to less evaluation preference for experiential purchases for individuals with low transcendence over enhancement values (mean − 1SD), *b* = −0.38, *t* = −4.19, *p* < 0.001. The negative association became weaker but still significant for individuals with the mean level of transcendence‐enhancement values, *b* = −0.18, *t* = −2.63, *p* = 0.009, and further became positive but nonsignificant for individuals with high transcendence‐enhancement values (mean + 1SD), *b* = 0.01, *t* = 0.08, *p* = 0.791. In other words, Chinese older adults with lower levels of self‐transcendence values tended to rate experiential purchases less favourably compared to their younger counterparts. Such age‐related differences in purchase preference became less pronounced and even reversed with increasing endorsement of self‐transcendence values.

**TABLE 3:** Age × transcendence‐enhancement interaction effects on purchase preference in Chinese and American participants, respectively.

Country	Variables	*B*	SE	*t*	*p*
China	Constant	0.47	0.07	6.50	0.000
	Current SES	0.21	0.07	3.26	0.001
	Childhood SES	0.03	0.07	0.41	0.684
	Age	−0.22	0.07	−3.19	0.002
	Transcendence‐enhancement	0.20	0.06	3.10	0.002
	Age × transcendence‐enhancement	0.18	0.06	3.25	0.001
United States	Constant	0.06	0.04	1.65	0.101
	Current SES	0.04	0.03	1.32	0.189
	Childhood SES	0.01	0.05	0.15	0.879
	Age	−0.07	0.03	−0.21	0.832
	Transcendence‐enhancement	0.20	0.05	4.12	0.000
	Age × transcendence‐enhancement	0.01	0.04	0.23	0.816

*Note:* Age and transcendence‐enhancement were standardised.

In American participants, while transcendence‐enhancement values still had a significant positive main effect on evaluation preference for experiential purchases, *b* = 0.20, *t* = 4.12, *p* < 0.001, both the main effect of age, *b* = −0.07, *t* = −0.21, *p* = 0.832, and the age × transcendence‐enhancement interaction, *b* = 0.01, *t* = 0.23, *p* = 0.816, were nonsignificant.

### 3.2 Sociocultural Context, Openness‐Conservation Values and Age on Purchase Preference

Table 4 presented the multiple regression model testing how sociocultural context and the openness‐conservation dimension of personal values interacted with age to influence purchase preference. Again, there was a significant positive main effect of openness‐conservation on evaluation preference towards experiential purchases, *b* = 0.27, *t* = 6.39, *p* < 0.001. There was also a significant positive age × openness‐conservation interaction, *b* = 0.26, *t* = 6.77, *p* < 0.001, indicating that the association between age and preference for experiential purchases was more positive for individuals with stronger endorsement of the openness values. Similarly, supporting Hypothesis 5, there was a significant country × age × openness‐conservation interaction, *b* = −0.34, *t* = −3.95, *p* < 0.001, indicating that sociocultural context further moderated the age × openness‐conservation interaction.

**TABLE 4:** Country × age × openness‐conservation interaction effects on purchase preference.

Variables	*B*	SE	*t*	*p*
Constant	0.65	0.05	12.70	0.000
Current SES	0.02	0.04	0.61	0.544
Childhood SES	−0.01	0.04	−0.35	0.727
Age	−0.04	0.05	−0.73	0.464
Country (0 = China, 1 = United States)	−0.63	0.08	−8.32	0.000
Openness‐conservation	0.27	0.04	6.39	0.000
Country × age	0.05	0.07	0.69	0.489
Country × openness‐conservation	−0.15	0.09	−1.69	0.092
Age × openness‐conservation	0.26	0.04	6.77	0.000
Country × age × openness‐conservation	−0.34	0.09	−3.95	0.000

*Note:* Age and openness‐conservation were standardised.

**FIGURE 2:**
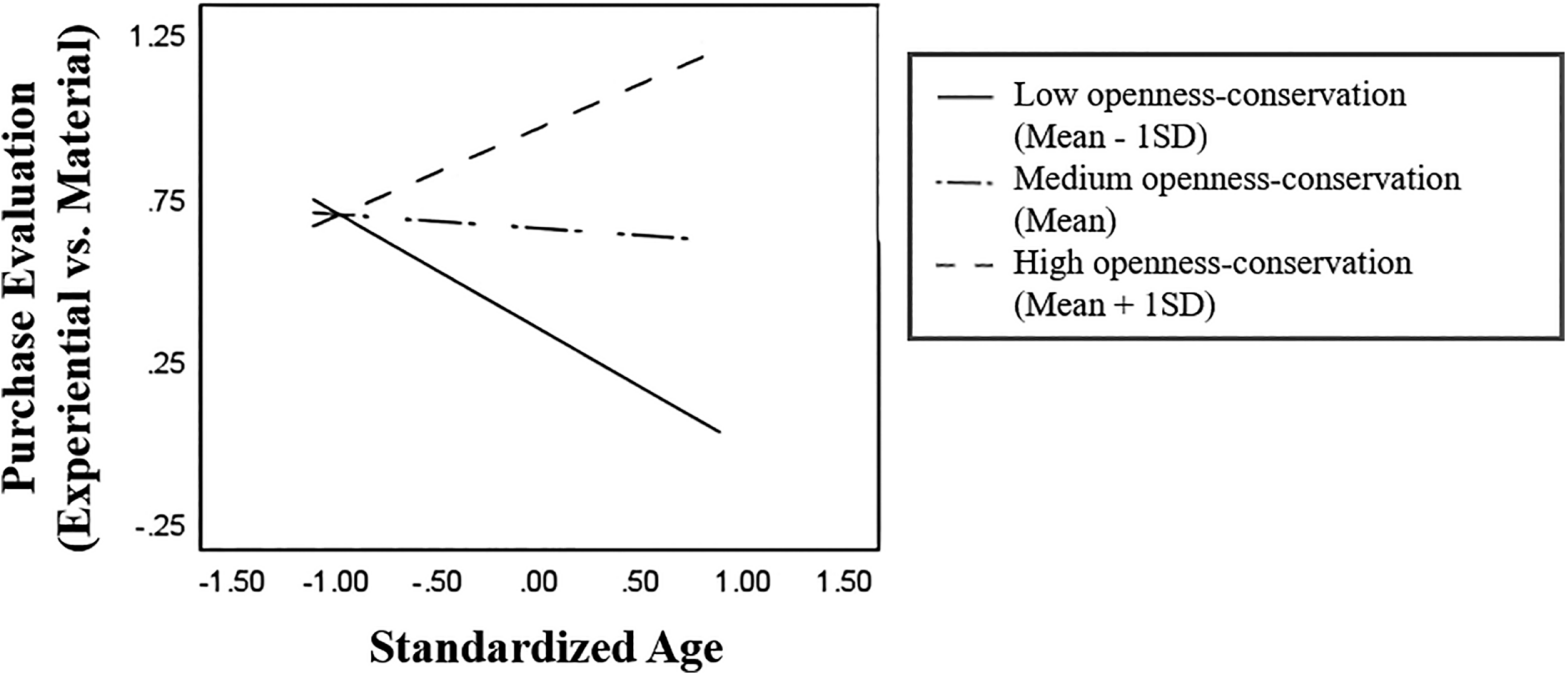
Simple slope analyses illustrating the age × openness‐conservation interaction on purchase preference in Chinese participants.

We further examined how the openness‐conservation values interacted with age to influence purchase evaluation preference in Chinese and American participants, respectively (see Table 5). For Chinese participants, while the main effect of age was not significant (*b* = −0.05, *t* = −0.72, *p* = 0.474), the openness‐conservation values had a significant positive main effect (*b* = 0.25, *t* = 4.66, *p* < 0.001) on purchase preference. Notably, the interaction between age and openness‐conservation values was significant, *b* = 0.26, *t* = 5.25, *p* < 0.001. Simple slope analyses (see Figure 2) showed that for individuals with low openness‐to‐change over conservation values (mean − 1SD), age was significantly related to less evaluation preference for experiential purchases, *b* = −0.36, *t* = −4.06, *p* < 0.001. The negative association became weaker and nonsignificant for individuals with the mean level of openness‐conservation values, *b* = −0.04, *t* = −0.68, *p* = 0.500, and further became positive and significant for individuals with high openness‐conservation values (mean + 1SD), *b* = 0.27, *t* = 3.13, *p* = 0.002. In other words, Chinese older adults with lower levels of openness‐to‐change values tended to rate experiential purchases less favourably than younger individuals. However, with the increasing endorsement of openness‐to‐change values, the age‐related difference in purchase preference became less apparent and may even reverse.

**TABLE 5:** Age × openness‐conservation interaction effects on purchase preference in Chinese and American participants, respectively.

Country	Variables	*B*	SE	*t*	*p*
China	Constant	0.66	0.07	10.20	0.000
	Current SES	0.05	0.07	0.69	0.492
	Childhood SES	0.03	0.06	0.50	0.619
	Age	−0.05	0.06	−0.72	0.474
	Openness‐conservation	0.25	0.05	4.66	0.000
	Age × openness‐conservation	0.26	0.05	5.25	0.000
United States	Constant	0.05	0.04	1.37	0.172
	Current SES	0.02	0.04	0.54	0.586
	Childhood SES	−0.10	0.04	−2.18	0.030
	Age	0.00	0.03	0.07	0.946
	Openness‐conservation	0.14	0.05	2.85	0.005
	Age × openness‐conservation	−0.08	0.05	−1.57	0.118

*Note:* Age and openness‐conservation were standardised.

In American participants, while openness‐conservation values still had a significant positive main effect on evaluation preference to experiential purchases, *b* = 0.14, *t* = 2.85, *p* = 0.005, neither the main effect of age, *b* = 0.00, *t* = 0.07, *p* = 0.946, nor the age × openness‐conservation interaction, *b* = −0.08, *t* = −1.57, *p* = 0.118, was statistically significant.

## 4 Discussion

The current study provides important insights into age‐related differences in preference in purchase evaluations. Sociocultural context and personal values were found to jointly moderate the age‐related differences. In particular, Hypotheses 1 and 2 were supported in both Chinese and American participants. Stronger endorsement of transcendence over enhancement values and stronger endorsement of openness over conservation values were significantly related to higher evaluation preference for experiential over material purchases in both samples. The findings bridge earlier research on experiential advantages (e.g., van Boven and Gilovich 2003) with the consumer behaviour literature grounded in Schwartz's theory of personal values (e.g., Blomstervik and Olsen in press; Krystallis et al. 2012). Consistent with prior studies suggesting that experiential purchases are more related to the sense of social connectedness (Caprariello and Reis 2013; Kumar and Gilovich 2015; Yamaguchi et al. 2016), individuals prioritising self‐transcendence may find experiential purchases especially appealing due to their social and emotional benefits. Similarly, those who value openness‐to‐change may view experiential purchases as opportunities for novelty and growth, echoing findings that highlight the role of experiences in identity development and self‐actualisation (Carter and Gilovich 2012; Kim et al. 2016; Moldes et al. 2019).

Hypotheses 3 and 4 were only supported in Chinese participants. For Chinese individuals with low self‐transcendence or openness‐to‐change values, older age was associated with less evaluation preference towards experiential purchases. Conversely, for Chinese individuals who prioritise self‐transcendence or openness‐to‐change values, there was a more positive relationship between age and evaluation preference towards experiential purchases. As expected, individuals who prioritise social connections and others' well‐being and those who value novelty and new experiences are likely to be increasingly influenced by their personal values and prefer experiential over material purchases as they age.

However, no main effects of age or age × personal value interactions were observed in American participants. The results also supported Hypothesis 5. The age differences in purchase preferences and the age × personal value interactions were both more pronounced in China compared to the United States. The findings suggest that sociocultural contexts could play a key role in shaping personal values and purchase evaluations. Despite theoretical expectations of prioritising emotionally meaningful goals from socioemotional selectivity theory (Fung and Carstensen 2004), the motivational shifts with ageing may not universally translate into consumption preferences. Instead, the distinct sociocultural landscapes of China and the United States over recent decades may contribute to differences in how consumers of different age groups evaluate experiential and material purchases. In China, the rapid economic development could lead to changes in consumers' beliefs and behaviours, resulting in larger age‐related variations in purchase evaluations (Yao 2000). In contrast, the socioeconomic stability and relative cultural homogeneity in the United States over recent decades may contribute to more consistent consumer values and preferences across age groups (Hung et al. 2007).

The current study is a novel attempt to understand age‐related differences in evaluation preference towards experiential over material purchases, which is a critical research question facing the rapidly growing population of older adults across the globe. The findings suggest that there is no simple answer to this question. First, the sociocultural context could significantly influence the age‐related differences. As mentioned above, in Chinese participants, there tended to be a negative correlation between age and evaluation preference for experiential purchases, and significant interaction effects were observed between age and personal values on purchase evaluations. However, no significant age effects or age‐related interactions were found in American participants. The disparity in findings between Chinese and American participants could be attributed to the rapidly changing socioeconomic environment in China in recent years and the relatively stable socioeconomic conditions in the United States (Hung et al. 2007; Yao 2000).

Besides the sociocultural context, personal values also play important roles in moderating the age‐related differences in purchase evaluations. The stronger endorsement of transcendence over enhancement values and openness over conservation values was consistently related to a higher preference for experiential items in purchase evaluations in both Chinese and American participants. Moreover, both value dimensions moderated the association between age and purchase evaluation preferences in Chinese participants. Older Chinese individuals with lower levels of self‐transcendence or openness‐to‐change values exhibited less preference towards experiential purchases than younger adults. Conversely, older adults with higher levels of self‐transcendence or openness‐to‐change values are more likely to display a stronger preference towards experiential purchases than younger adults. As expected, individuals who prioritise social connections and others' well‐being and those who value novelty and new experiences are likely to be increasingly influenced by their personal values and prefer experiential over material purchases as they age.

The study provides theoretical contributions to the literature in the context of the silver economy and offers interdisciplinary innovations. At the cultural level, the findings highlight the importance of considering sociocultural context when examining age‐related trends in consumer behaviour. At the individual level, while previous research has revealed that purchase preference can be influenced by personal values such as autonomy (Li et al. 2022), this study extends the literature by applying Schwartz's (2012) more comprehensive framework of personal values. By integrating insights from developmental psychology, consumer research and Schwartz's (2012) value theory, this study depicts a more complete picture of how personal values and age interact to influence purchase preferences across adulthood in different cultural settings.

The current study has practical implications for the business sector as well. Facing the unprecedented population ageing trend, older consumers represent a growing and influential market segment (Hettich et al. 2018). By emphasising the complexity of age differences in purchase evaluations, the findings underscore the importance of considering personal values and sociocultural differences when investigating older consumers' purchase preferences and behaviours. Companies may adapt their marketing strategies and product development efforts for consumers of varying ages and backgrounds, thereby enhancing their ability to reach and engage the target customers.

We also acknowledge several limitations of the current study. First, due to the cross‐sectional nature of the study, it is challenging to differentiate whether the observed age differences in purchase evaluations stem from developmental changes or if they indicate different generations being shaped by value priorities in their upbringing contexts. Future investigations may replicate the study in a longitudinal design, tracking the evolution of individuals' purchase preferences over an extended period. Second, the study only focuses on the sociocultural differences between China and the United States. The findings may not be generalised to other countries. Future studies could extend the investigation to other countries and provide a more comprehensive understanding of how sociocultural context may influence the interplay between age and personal values in purchase evaluations. Third, the internal consistency of the openness‐conservation dimension is relatively low (*α* = 0.61), suggesting that the reliability of the measure may be limited. Nonetheless, the measurement invariance analyses confirmed the configural equivalence of the two‐factor model for the PVQ scale across the two samples, providing additional empirical evidence supporting the reliability of the measure. Meanwhile, metric equivalence was not supported for either the purchase evaluation measure or the PVQ scale, indicating that the weights of different items for corresponding purchase evaluations and personal values may vary across cultures. Although this may not affect the current results based on the arithmetic means of relevant items, future research could develop more culturally invariant tools to support more precise comparisons across cultures. Moreover, although we used widely adopted items to assess experiential versus material purchases, the two categories may not be mutually exclusive. Participants may interpret the same item (e.g., a meal at a nice restaurant) as either experiential or material, which could introduce variability in how purchases are perceived. Future research could add further clarification for the items and could examine factors contributing to individual differences in the perception of ambiguous items. Last, the study examined consumers' evaluations of experiential and material purchases. Although evaluations of the products can be a strong predictor of purchase decisions, they may not always align with actual purchase behaviours. Future research may further investigate age differences in actual purchase behaviours.

In conclusion, the current study provides insights about how sociocultural context and personal value moderate the age‐related differences in consumers' purchase evaluations. The findings highlight the potential ways in which age might influence consumers' perceptions and preferences through motivational shifts. Moreover, the findings reveal the underlying mechanisms behind age‐related differences in purchase evaluations by identifying the moderating role of personal values and sociocultural context. The study provides both theoretical and practical implications for understanding individuals' purchase preferences across adulthood.

## Author Contributions

**Haowei Peng:** methodology, data curation, formal analysis, writing – original draft, writing – review and editing, project administration. **Tianyuan Li:** conceptualization, methodology, data curation, formal analysis, supervision, funding acquisition, writing – original draft, writing – review and editing, project administration.

## Funding

This work was supported by the Basic and Applied Basic Research Foundation of Guangdong Province (2023A1515110295).

## Ethics Statement

This study was approved by the Applied Psychology Institutional Review Board of the Chinese University of Hong Kong, Shenzhen (EF20220708001) on 13 August 2022.

## Consent

Informed consent was obtained from all individual participants included in the study.

## Conflicts of Interest

The authors declare no conflicts of interest.

## Data Availability

The data that support the findings of this study are available from the corresponding author upon reasonable request.
